# Genetic dissection of drought and heat‐responsive agronomic traits in wheat

**DOI:** 10.1111/pce.13577

**Published:** 2019-06-24

**Authors:** Long Li, Xinguo Mao, Jingyi Wang, Xiaoping Chang, Matthew Reynolds, Ruilian Jing

**Affiliations:** ^1^ National Key Facility for Crop Gene Resources and Genetic Improvement/Institute of Crop Sciences Chinese Academy of Agricultural Sciences Beijing 100081 China; ^2^ International Maize and Wheat Improvement Center Texcoco 56237 Mexico

**Keywords:** abiotic stress tolerance, agronomic traits, biparental QTL mapping, drought stress, genome‐wide association study, haplotype block, heat stress, heritable covariation, linkage disequilibrium, wheat

## Abstract

High yield and wide adaptation are principal targets of wheat breeding but are hindered by limited knowledge on genetic basis of agronomic traits and abiotic stress tolerances. In this study, 277 wheat accessions were phenotyped across 30 environments with non‐stress, drought‐stressed, heat‐stressed, and drought‐heat‐stressed treatments and were subjected to genome‐wide association study using 395 681 single nucleotide polymorphisms. We detected 295 associated loci including consistent loci for agronomic traits across different treatments and eurytopic loci for multiple abiotic stress tolerances. A total of 22 loci overlapped with quantitative trait loci identified by biparental quantitative trait loci mapping. Six loci were simultaneously associated with agronomic traits and abiotic stress tolerance, four of which fell within selective sweep regions. Selection in Chinese wheat has increased the frequency of superior marker alleles controlling yield‐related traits in the four loci during past decades, which conversely diminished favourable genetic variation controlling abiotic stress tolerance in the same loci; two promising candidate paralogous genes colocalized with such loci, thereby providing potential targets for studying the molecular mechanism of stress tolerance–productivity trade‐off. These results uncovering promising alleles controlling agronomic traits and/or multiple abiotic stress tolerances, providing insights into heritable covariation between yield and abiotic stress tolerance, will accelerate future efforts for wheat improvement.

## Abstract 2

We uncovered consistent marker alleles for agronomic traits across different environments and eurytopic marker alleles controlling multiple abiotic stress tolerances in wheat. Selective sweep analysis showed that the gradual loss of advantageous variation for abiotic stress tolerances due to preferential selection for yield potential may be a common phenomenon in current wheat breeding. Two promising candidate genes identified in the present study provide potential targets for studying the molecular mechanism of stress tolerance‐productivity trade‐off. These results are timely in terms of developing efficient molecular markers and dissection of the functions of relevant genes for wheat improvement in times of unexpected climatic fluctuations.

**Abbreviations**
DHS: drought‐heat‐stressed
DS: drought‐stressed
DTF: day to flowering
ESNP: effective spikes number per plant
GNP: grain number per main spike
GWAS: genome‐wide association study
GYP: grain yield per plant
HS: heat‐stressed
NS: non‐stress
PH: plant height
SL: main spike length
SMA: superior marker alleles
SNPP: spikelet number per main spike
TKW: thousand kernel weight

## 1 INTRODUCTION

Wheat (*Triticum aestivum* L.) is one of the three major crops providing about 19% of global dietary energy (Ray, Mueller, West, & Foley, 2013). Given the increasing demand of global food supply and frequent episodes of abiotic stresses (water deficit and heat stress), producing wheat cultivars with higher yield potential as well as drought and heat tolerances is required (Godfray et al., 2010; Tilman, Balzer, Hill, & Befort, 2011). Molecular breeding has been proposed to be an effective and powerful strategy for crop improvement (Langridge & Reynolds, 2015; Qian, Guo, Smith, & Li, 2016) but requires a better understanding of relevant genetic architecture. Therefore, dissecting the genetic basis of agronomic traits and responses to drought and heat stresses is indispensable for the improvement of wheat.

Genome‐wide association study (GWAS) is a powerful tool for dissecting genetic basis of agronomic traits and has been successfully applied for discovering relevant genes in rice and maize (Pace, Gardner, Romay, Ganapathysubramanian, & Luebberstedt, 2015; Yano et al., 2016). Because of genomic complexity and high proportion of repetitive sequences, application of GWAS in wheat lags behind when compared with other major crops. Most of the quantitative trait loci (QTL) controlling agronomic traits in wheat were present as genetic positions (Schulthess et al., 2017; Sukumaran, Reynolds, & Sansaloni, 2018). Recent advances in accurate resequencing and development of single nucleotide polymorphism (SNP) array are expected to solve this problem and revolutionize GWAS in wheat (Cui et al., 2017; IWGSC, 2018). In addition, most of the reported QTL controlling agronomic traits were identified under high‐yield potential conditions (Mora et al., 2015; Sukumaran, Dreisigacker, Lopes, Chavez, & Reynolds, 2015; Sun et al., 2017), and relatively few studies have examined consistency of QTL across contrasting environments including stress and non‐stress (NS; Pinto et al., 2010; Sukumaran et al., 2018). Therefore, identifying stable QTL/alleles for better agronomic performance under diverse environments is essential for dealing with the changing climate.

Drought and heat are two major abiotic stresses constraining global wheat production (Tricker, Elhabti, Schmidt, & Fleury, 2018) and are more likely to occur simultaneously rather than separately at late growth stages of wheat in semi‐arid and hot‐growing regions (Olesen et al., 2011; Pradhan, Prasad, Fritz, Kirkham, & Gill, 2012). Several studies found that the combination of drought and heat stress had greater negative effect on the productivity of crops compared with each of the different stresses applied individually (Prasad, Pisipati, Momcilovic, & Ristic, 2011). To date, various QTL/alleles responding to drought or heat stress were detected in wheat (Kulkarni et al., 2017; Maulana et al., 2018), whereas studies of the genetic basis of responses to combined drought and heat stress are rare (Liu et al., 2019). Previous studies revealed that the responses of plants to a combined abiotic stresses are unique and cannot be directly predicted from the plant performance under different stresses individually (Suzuki, Rivero, Shulaev, Blumwald, & Mittler, 2014), and most of the transcriptome changes responding to combined heat and drought stress differ from that under individual stressor (Sakuma et al., 2006). Along with the increasing drought stress induced by global warming (Dai, 2013), the identification of QTL/alleles that confer tolerance to drought, heat, and the dual stresses have been regarded as one of the most important task for genetic research in wheat (Lobell, Sibley, & Ivan Ortiz‐Monasterio, 2012; Nevo & Chen, 2010).

Both yield potential and abiotic stress tolerance are crucial for wheat improvement, but simultaneously improving these two kinds of traits is a great challenging for crop breeding. It is partly because many complex traits tend to be tightly integrated or controlled by pleiotropic genes, resulting in heritable covariation (Fang et al., 2017). One noteworthy example is the function of the SWI2/SNF2 chromatin remodelling ATPase BRAHMA (BRM) in the balance between growth and drought stress tolerance in *Arabidopsis* (Han et al., 2012). However, knowledge of the genetic basis of trade‐off between yield and stress tolerance is very limited in crops. Wheat cultivars are mainly achieved through conventional breeding methodologies with the goal of achieving increments in yield potential and often maintaining disease resistance. It is not clear whether the selection for yield potential facilitates the genetic improvement for stress tolerance. Therefore, identification and characterization of QTL/alleles regulating the stress tolerance–productivity trade‐off are essential to further understand the heritable covariation between high yield and yield stability in wheat and to further enable researchers and breeders to determine applicable selection strategies for various ecological regions.

In this study, four wheat collections were genotyped with high‐density wheat 660K SNP array, of which 277 wheat accessions and 150 doubled haploid lines were cultivated in the field under non‐stress (NS), drought‐stressed (DS), heat‐stressed (HS), and drought‐heat‐stressed (DHS) conditions and phenotyped for eight agronomic traits. Our study aimed to (a) identify stable genetic variations controlling agronomic traits under NS and abiotic (drought and/or heat) stress conditions; (b) identify genetic variations underlying responses for drought, heat and dual stresses; and (c) explore the heritable covariation between yield potential and abiotic stress tolerance, and the variations caused by artificial selection.

## 2 MATERIALS AND METHODS

### 2.1 Plant materials

Four wheat collections were used in this study. Collection 1 consisted of 277 winter wheat accessions, most of whose flowering dates occurred within 1 week; the collection contained 12 landraces, 35 advanced lines, and 230 modern cultivars, mainly planted in the Yellow and Huai River Valleys Facultative Wheat Zone and Northern Winter Wheat Zone in China (Supporting Information Table S1); and GWAS was applied to the collection. Collection 2 was comprised of 150 doubled haploid lines, which were derived from two Chinese winter wheat cultivars, Hanxuan 10 (a variety with drought and heat stress tolerance) and Lumai 14 (a variety with high yield; Liu, Li, Chang, & Jing, 2013). This collection was applied to linkage‐based QTL mapping. Collections 3 and 4 are derived populations, contained 102 and 87 cultivars derived from founder genotypes Xiaobaimai and Mazhamai, two landraces originated in the Northern Winter Wheat Zone and Yellow and Huai River Valleys Facultative Wheat Zone in China, respectively (Supporting Information Table S2), and these two collections were employed to perform selective sweep analysis.

### 2.2 Growth conditions and phenotyping

Collections 1 and 2 were planted in 30 different environments (year × site × treatment) with NS, DS, HS, and DHS treatments (Supporting Information Table S3). The NS treatment was thrice irrigated with 750 m^3^ ha^−1^: before winter, at booting and flowering when the amount of rainfall was insufficient. The DS treatment was represented by rainfed condition, and the rainfalls in the growing seasons of different years and sites are summarized in Table S3. The HS treatment was simultaneously irrigated with NS group, and the heat treatment applied using thermal stress shelters, which were built when all accessions were about 1‐week post‐anthesis, by covering plastic film on steel frames over the trial plots (Supporting Information Figure S1). The DHS treatment was carried out by combining the rainfed and thermal stress treatments described above. The temperature and soil water content under different environments, together with comparative results using the *t* test, are shown in Supporting Information Figure S2. Each experimental plot was 2 m in length with four rows and a row spacing of 30 cm, with 40 seeds per row. Field management was consistent with local practices for wheat production. Five healthy individuals selected from the middle of the two internal rows in each plot were used to investigate grain yield per plant (GYP), effective spike number per plant (ESNP), spikelet number per main spike (SNPP), grain number per main spike (GNP), thousand kernel weight (TKW), plant height (PH), and main spike length (SL) according to established protocols (Pask, Pietragalla, Mullan, & Reynolds, 2012); days to flowering (DTF) was assessed as the interval between the date of sowing and the date at which 50% of spikes per plot have extruded at least one anther. The traits investigated under each treatment were represented by the suffix “‐treatment” in the present study, for instance, GYP‐DS denotes grain yield per plant under drought stress. Given the heat stress was applied post‐anthesis, the SNPP, PH, SL, and DTF under HS and DHS were excluded for subsequent data analyses because that these traits have already settled at this stage.

### 2.3 Statistical analysis of phenotypic data

Analysis of variance (anova) was performed to test the effect of genotype (G), treatment (T), and combined year and site (YS) with their interactions based on combined linear mixed model using the software IciMapping 4.1. In addition, broad‐sense heritabilities for each trait under different treatments was calculated across environments (years and sites) as presented by Piepho and Moehring (2007). Best linear unbiased predictions (BLUPs) of investigated traits under each treatment for an individual genotype were estimated across years and sites using lme4 package in R 3.3.0. Here, genotypes, years, and sites were assumed as fixed factors, whereas environments, replicates, and error terms were considered as random factors. Correlation among the different traits was evaluated based on the Pearson correlation coefficients between BLUPs using IBM SPSS 19.0. Genotype tolerances to drought and/or heat stresses for each trait were evaluated using relative value (Guo et al., 2018), represented by the suffix “‐R” in the study, and calculated as the ratio of the BLUPs of investigated traits in stress conditions to that in NS condition; for instance, GYP‐DS‐R denotes the drought stress tolerance based on GYP, and GNP‐HS‐R denotes the heat stress tolerance based on grain number per spike.

### 2.4 Genotyping

Genomic DNA of all accessions in the four collections was extracted according to the protocol of Ogbonnaya et al. (2001), DNA quantity was measured spectrophotometrically, and DNA integrity was confirmed on agarose gel. Genotyping was performed on the wheat 660K SNP array by Capital Bio Corporation (http://www.capitalbio.com) using the Affymetrix GeneTitan System according to the Axiom 2.0 Assay Manual Workflow protocol. Wheat 660K SNP array consisted of 630 517 SNPs developed from the transcriptome and genome sequencing. Allele calling was carried out using the Affymetrix proprietary software according to Axiom Best Practices Genotyping Workflow. The physical locations of SNPs were identified based on the IWGSC wheat genome sequence (IWGSC RefSeq v1.0).

### 2.5 Genome‐wide association study (GWAS)

After removing nucleotide variations with missing rates ≥20% and minor allele frequency (<0.05), 395 681 SNPs were used for GWAS in the present study. The population structure of the Collection 1 was analysed using software STRUCTURE 2.3.4. Principal component analysis was performed using GCTA software. Linkage disequilibrium (LD) was estimated as the squared allele frequency correlation using −*r* ^2^ command in the software PLINK 1.9. LD decay graph was plotted according to LD of SNP pairs within 100 kb, and step size was set to 50 kb. Two GWAS methods, that is, single‐locus and multilocus analysis, were used to detect the marker–trait associations. We performed single‐locus analysis using TASSEL 5.0 based on general linear model (GLM) and mixed linear model (MLM) as follows:

$$Y_{\mathrm{GLM}}=\mathrm{X\alpha}+\mathrm{Q\beta}+e,$$

$$Y_{\mathrm{MLM}}=\mathrm{X\alpha}+\mathrm{Q\beta}+\mathrm{K\mu}+e,$$

where *Y* is the vector of phenotype, *X* is the vector of marker genotypes; *Q* is the principal components matrix (represented by the first five principal components); *K* is the relative kinship matrix; *α*, *β*, and *μ* are the corresponding effects; and *e* is a matrix of residual effects. *X* and *Q* matrix were fitted as fixed effects, and *K* and *e* matrix were fitted as random effects. In addition, to correct the confounding effects of causal loci, a multilocus GWAS based on fixed and random model Circulating Probability Unification (FarmCPU) was also conducted in the present study, which allows a fixed effect model and a random effect model to perform iteratively to improve statistical power and computational efficiency (Liu, Huang, Fan, Buckler, & Zhang, 2016). Given the non‐independence of SNPs due to LD, a modified Bonferroni correction described by Li, Yeung, Cherny, and Sham (2012) was used to determine the genome‐wide significance thresholds (*p* < 1.88 × 10^−5^) of the GWAS. Haplotype blocks were determined base on the interpretation of the block definition suggested by Gabriel et al. (2002) using Plink 1.9.

### 2.6 Biparental QTL mapping

By screening polymorphic markers with low missing rate (≤20%), a total of 114 545 SNPs were available for Collection 2; together with 224 SSR markers mapped previously by Hao et al. (2003), a linkage map, which is 4082.4 centimorgans in length and contains 1854 bins with 2.2 cM per bin on average, was constructed using JoinMap 4.0 (Supporting Information Table S4). Detection of QTL was performed by the inclusive composite interval mapping method (Li, Ribaut, Li, & Wang, 2008) using the software IciMapping 4.1 with a 0.1‐cM walking speed, and the LOD threshold was set to 2.5 based on the results of 1000 permutations.

### 2.7 Bioinformatics analysis

Reported QTL were retrieved from PubMed database; the physical location of these QTL was obtained from URGI database. Putative genes near to significant SNPs in haplotype blocks were obtained according to IWGSC RefSeq v1.1 annotation, and the gene expression data of candidate genes were downloaded from the Wheat Expression Browser. *F*‐statistics (*F*_ST_) is an index of population differentiation based on genetic polymorphism; the *F*_ST_ between accession groups of different eras or generations in Collections 1, 3, and 4 were evaluated using a 100‐kb sliding window with 50‐kb steps; and genomic regions with top 5% *F*_ST_ were defined as potential selective sweeps.

## 3 RESULTS

### 3.1 Variations of agronomic traits between experiments depended on temperature and soil moisture.

**Figure 1:**
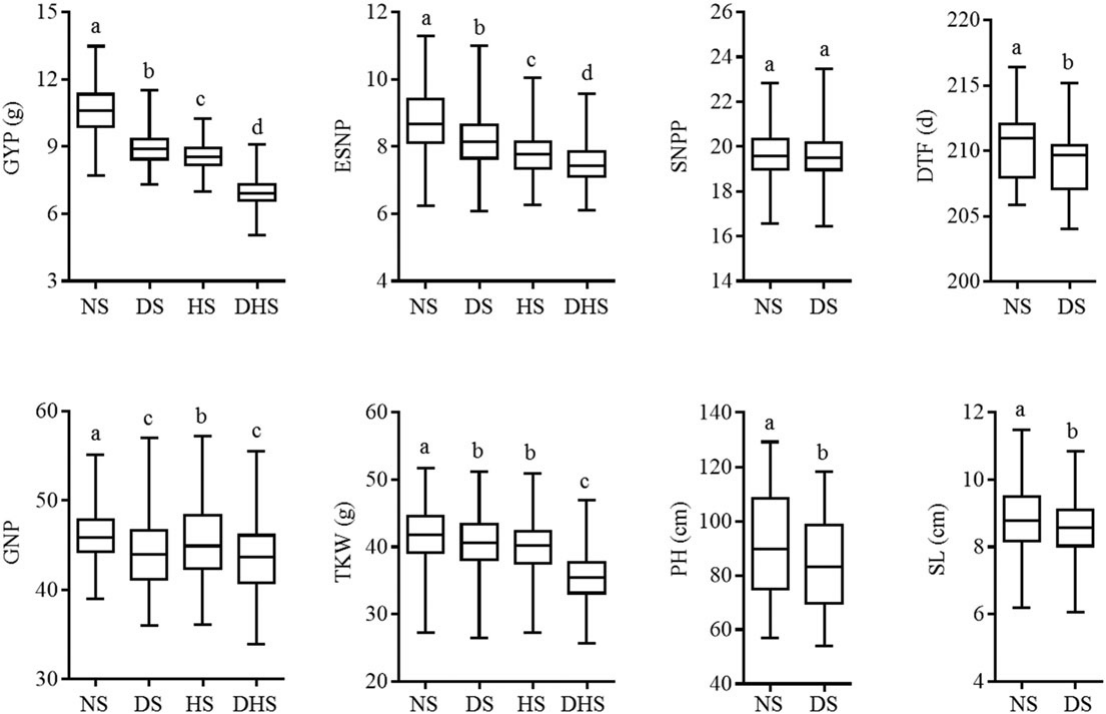
Boxplots of the best linear unbiased predictions of the agronomic traits of 277 accessions in Collection 1. DHS: drought‐heat‐stressed; DS: drought‐stressed; DTF: day to flowering; ESNP: effective spikes number per plant; GNP: grain number per main spike; GYP: grain yield per plant; HS: heat‐stressed; NS: non‐stress; PH: plant height; SL: main spike length; SNPP: spikelet number per main spike; TKW: thousand kernel weight. Different letters indicate statistically significant differences at the level of *p* < .01

anova was performed to divide the variation in genotype (G), treatment (T), and combined year and site (YS) components and their interactions (Supporting Information Table S5). The variation in agronomic traits differed significantly between genotypes (G; *p* < .01), treatments (except SNPP; T; *p* < .01), and combined years and locations (except TKW; YS; *p* < .01). Further, the agronomic traits except ESNP and DTF of each genotype responded differently to combined years and sites (G × YS; *p* < .01). GYP was moderately heritable with broad‐sense heritability (*H* ^2^) of 0.58, 0.55, 0.54, and 0.48 under NS, DS, HS, and DHS, respectively, and other agronomic traits except ESNP under DHS (0.45) had higher heritabilities than GYP. The range of phenotypic variation differed among traits; the highest coefficient of variation (*CV*) was observed for PH‐NS (20.1%), followed by PH‐DS (19.6%); and the lowest coefficient of variation was observed for DTF‐NS (1.3%), followed by DTF‐DS (1.4%). More descriptive statistics of the investigated traits are summarized in Supporting Information Table S6. The agronomic traits showed significant differences in NS and stress conditions except for SNPP (Figure 1). In addition, the average BLUPs of GYP and ESNP under DS are significant higher than that of HS, whereas the average BLUP of GNP‐DS is significantly lower than that of GNP‐HS; none significant difference was observed between TKW‐HS and TKW‐DS. These results suggest that drought stress represented by rainfed condition in the present study has more negative influence on the seed formation of main spikes compared with terminal heat stress, and the latter may limit the development of young spikes in a greater degree than the former, leading to lower ESNP and GYP. The lowest BLUPs of agronomic traits were observed under DHS, indicating that the greatest reduction of yield is driven by combined drought and heat stress.

### 3.2 Phenotypic trait correlations

**Figure 2:**
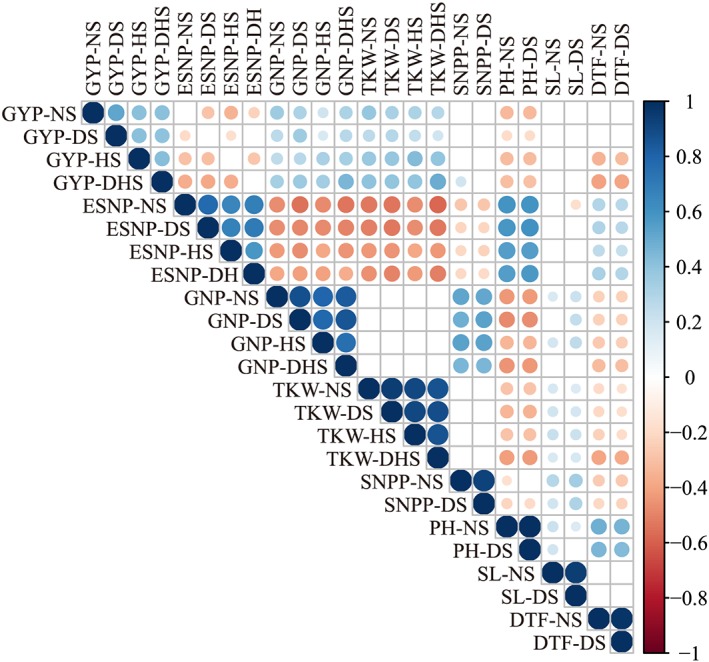
Heat map of the correlations between the best linear unbiased predictions of the agronomic traits. DHS: drought‐heat‐stressed; DS: drought‐stressed; DTF: day to flowering; ESNP: effective spikes number per plant; GNP: grain number per main spike; GYP: grain yield per plant; HS: heat‐stressed; NS: non‐stress; PH: plant height; SL: main spike length; SNPP: spikelet number per main spike, TKW: thousand kernel weight

Significant correlations were observed among the same trait under different treatments (Figure 2). GYP was positively correlated (*p* < .01) with GNP and TKW but was negatively correlated (*p* < .01) with ESNP and PH across treatments; non‐significant (*p* > .01) correlations of GYP were found with SNPP and SL. We also observed that GYP‐HS and GYP‐DHS were negatively correlated (*p* < .01) with DTF, suggesting that earlier initiation of grain‐filling is beneficial in reducing the losses of grain yield caused by heat stress at later growth periods. In addition, ESNP and PH was positively correlated (*p* < .01), but both of them were negatively correlated (*p* < .01) with GNP, TKW, and SNPP across treatments, which suggest the trade‐off between vegetative and reproductive growth.

### 3.3 Population structure, LD, and haplotype block analysis

**Figure 3:**
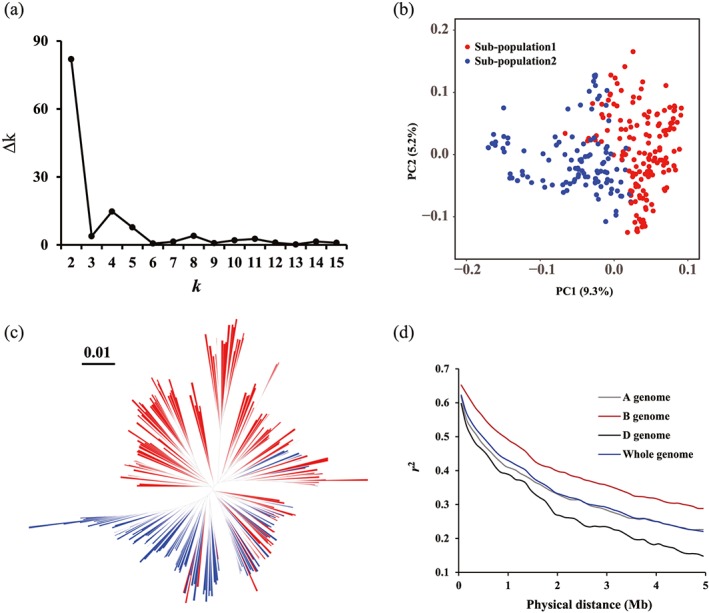
Analysis of the population structure and linkage disequilibrium decay of Collection 1 consisted of 277 accessions. (a) Plot of Δ*k* against putative *k* ranging from 2 to 15. (b) Plot of first principle component (PC1) against second principle component (PC2). (c) Neighbour‐joining phylogenetic tree, colour‐coded from the STRUCTURE results. (d) Linkage disequilibrium decay for the whole genome and sub‐genomes [Colour figure can be viewed at http://wileyonlinelibrary.com]

Based on the STRUCTURE algorithm, the second‐order likelihood (∆*k*) was calculated and showed a peak value when the given *k* (the number of subpopulations based on the model) is two (Figure 3a). Accordingly, the best possible numbers of subpopulations was considered as two. Furthermore, the results of principal component and neighbour‐joining tree analysis displayed a similar pattern of relationships among the 277 accessions estimated by STRUCTURE (Figures 3b and 3c). LD (indicated by *r* ^2^) dropped to 0.5 at 450 kb for whole genome (Figure 3d) but with variations among different sub‐genomes. The LD extent in D genome was 250 kb, similar to that of A genome (350 kb), but smaller than B genome (850 kb). Further, we performed haplotype block analysis using 1000 kb as maximum limitation, and a total of 24 103 haplotype blocks were detected on the whole genome; the highest number of haplotype block was detected on A genome (8808), followed by B genome (9508) and D genome (5787). In addition, 89.2% (353 120/395 682) of the SNPs were involved in these haplotype blocks, indicating broad block coverage (Supporting Information Table S7).

### 3.4 GWAS revealed consistent marker–trait associations for agronomic traits

**Figure 4:**
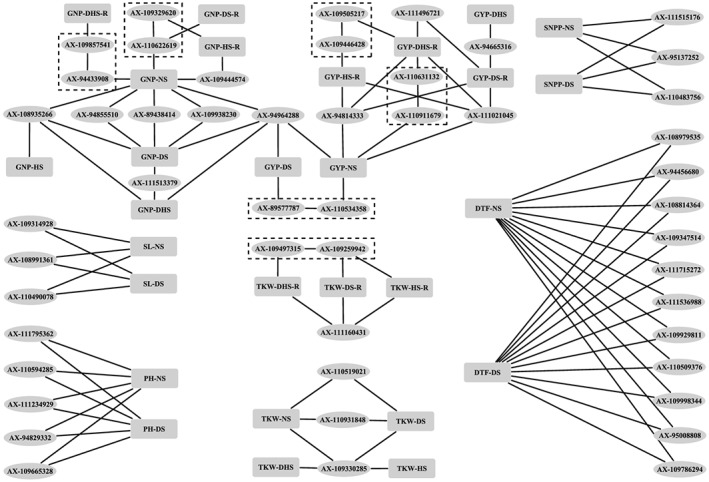
Consistent candidate SNPs for agronomic traits and abiotic stress tolerances. The dashed boxes indicate shared haplotype blocks

The single‐locus GWAS based on GLM and MLM and multilocus GWAS based on FarmCPU were conducted to detect the marker–trait associations for agronomic traits under different treatments. Given the trade‐off between dismissing false positives and accepting false negatives (Wen et al., 2018), the SNPs, which exceeded the significant threshold (*p* < 1.88 × 10^−5^) in at least two models, were regarded as associated SNP in the present study, and the peak SNPs (SNP with minimum *p*‐value) for each loci were defined as candidate SNP (cSNP). In total, 189 cSNPs were identified for agronomic traits. To be specific, 109, 69, 22, and 26 cSNPs were identified under NS, DS, HS, and DHS, respectively (Supporting Information Table S8). Individually, 34 cSNPs were identified for GYP, the highest number of GYP‐associated cSNPs was identified under NS (19), followed by DHS (8), HS (7), and DS (4); one cSNP (AX‐94964288) on chromosome 2B associated with both GYP‐NS and GYP‐DS. Moreover, a GYP‐NS‐associated cSNP (AX‐89577787) and a GYP‐DS‐associated cSNP (AX‐110534358) located on the same haplotype block (6D, 469047793‐469170884 bp), the LD (denoted by *r* ^2^) between these two cSNPs was 0.892. For ESNP, 6, 8, 3, and 3 cSNPs were identified under NS, DS, HS, and DHS, respectively, but no cSNP was simultaneously identified under different environments. For TKW, 6, 7, 5, and 4 cSNPs were identified under NS, DS, HS, and DHS, respectively, and three cSNPs (AX‐110519021, AX‐110931848, and AX‐109330285) on chromosome 2A, 5A, and 6B simultaneously associated with TKW under NS and DS, of which AX‐109330285 was also associated with TKW under HS and DHS, suggesting a consistent effect on TKW under multiple environments. For GNP, 38, 8, 7, and 11 cSNPs were identified under NS, DS, HS, and DHS, respectively. Five cSNPs (AX‐108935266, AX‐94855510, AX‐89438414, AX‐109938230, and AX‐94964288) on chromosome 1A, 2B, 3D, 4A, and 4B associated with both GNP‐NS and GNP‐DS, of which AX‐94964288 was also associated with GNP‐DHS, remarkably, AX‐108935266 associated with GNP under four treatment conditions. Moreover, AX‐94964288 was simultaneously associated with GYP‐NS and GYP‐DS as mentioned above, suggesting that the causal gene indicated by this cSNP may control GNP, hence affect the GYP. For GNPP, PH, SL, and DTF, 7, 8, 6, and 20 cSNPs were identified under NS, and 7, 8, 5, and 23 cSNPs were identified under DS, and 3, 5, 3, and 11 cSNPs separately associated with GNPP, PH, SL, and DTF were identified under both NS and DS. More information of the consistent cSNPs is summarized in Figure 4.

### 3.5 GWAS revealed marker–trait associations for drought and/or heat stress tolerance

A total of 65, 27, and 30 cSNPs were identified for trait‐DS‐R (representing drought stress tolerance), trait‐HS‐R (representing heat stress tolerance), and trait‐DHS‐R (representing dual stress tolerance), respectively. To be specific, 20, 14, and 19 cSNPs were identified for GYP‐DS‐R, GYP‐HS‐R, and GYP‐DHS‐R, respectively. Among which, AX‐111496721 on chromosome 2B was simultaneously associated with GYP‐DS‐R and GYP‐DHS‐R, and a GYP‐HS‐R‐associated cSNP (AX‐109446428) located on the same haplotype block (6B, 189131057–189527778) with a GYP‐DHS‐R‐associated cSNP (AX‐109505217); the LD between these two cSNPs was 0.955. Moreover, AX‐94814333 (6A) and AX‐111021045 (6B) were associated with multiple stress tolerances (GYP‐DS‐R, GYP‐HS‐R, and GYP‐DHS‐R). Eight, eight, and three cSNPs were associated with GNP‐DS‐R, GNP‐HS‐R, and GNP‐DHS‐R, respectively. A GNP‐DS‐R‐associated cSNP (AX‐110622619) and a GNP‐HS‐R‐associated cSNP (AX‐109329620) co‐located on the same haplotype block (5B, 686049751–686895826), whereas no significant associated SNPs were detected for GNP‐DHS‐R in this haplotype block, suggested that genetic dissection of combined heat and drought stress tolerance cannot be performed directly by studying plant response when heat and drought stresses were applied separately. Three, two, and six cSNPs were identified for TKW‐DS‐R, TKW‐HS‐R, and TKW‐DHS‐R, respectively. Among which, AX‐111160431 was simultaneously associated with TKW‐DS‐R, TKW‐HS‐R, and TKW‐DHS‐R. Furthermore, AX‐109259942, which associated with TKW‐DS‐R and TKW‐HS‐R, located on haplotype block (2D, 649592783–649782622), also harboured a TKW‐DHS‐R‐associated cSNP (AX‐109497315). In addition, 5, 26, and 1 cSNPs were identified for GNPP‐DS‐R, PH‐DS‐R, and SL‐DS‐R, respectively. More information of the cSNPs were summarized in Figure 4 and Table S8. Noteworthy, we also observed six loci on chromosomes 4A, 5B, 6A, 6B, and 6D simultaneously associated with abiotic stress tolerances and corresponding agronomic traits under NS. For example, AX‐94814333, which is the common cSNP for GYP‐DS‐R, GYP‐HS‐R, and GYP‐DHS‐R, simultaneously associated with GYP‐NS (1.26 × 10^−5^); and a GNP‐DHS‐R‐associated cSNP (AX‐109857541) significantly associated with GNP‐NS (1.26 × 10^−7^), located on the same haplotype block (4A, 681177122–681659340 bp) with GNP‐NS‐associated cSNP (AX‐94433908). However, the allelic effect of these SNPs showed clear inversion of effects for abiotic stress tolerance and corresponding agronomic traits under NS (Supporting Information Table S8). For example, AX‐94814333 had positive effect on GYP‐DS‐R (0.04), GYP‐HS‐R (0.08), and GYP‐DHS‐R (0.02), but negative effect on GYP‐NS (−0.28 g), suggesting a heritable covariation between yield potential and abiotic stress tolerances.

### 3.6 Validation of GWAS signals

**Figure 5:**
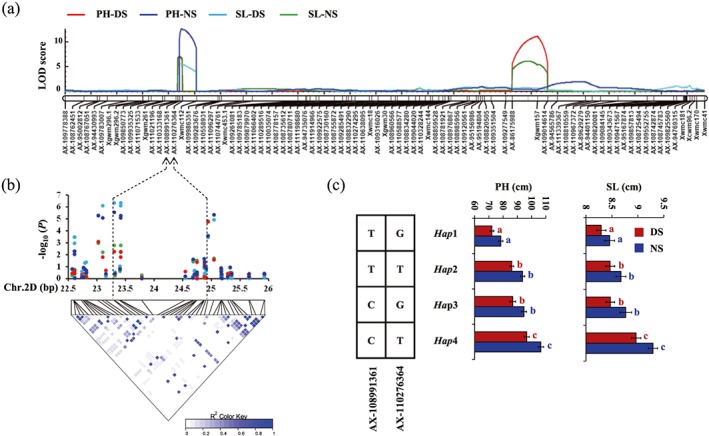
Co‐localization of consistent candidate SNPs (cSNPs) and quantitative trait loci for PH and SL. (a) Linkage mapping for PH and SL on chromosome 2D. (b) Local Manhattan plot (top) and linkage disequilibrium heatmap (bottom) surrounding the cSNPs for PH and SL on chromosome 2D. The dotted arrows indicate the position of cSNPs (AX‐108991361 and AX‐110276364). (c) Boxplots for PH and SL based on the haplotypes (*Hap*), which formed by the variation of the marker allele in AX‐108991361 and AX‐110276364. Different letters indicate statistically significant differences at the level of *p* < .01. PH: plant height; SL: main spike length [Colour figure can be viewed at http://wileyonlinelibrary.com]

To further validate the reliability of the GWAS results, we phenotyped 150 DH lines under 10 environments and performed QTL mapping analyses. A total of 135 and 46 QTL for agronomic traits and stress tolerance were identified, respectively (Supporting Information Table S9), and further projected into wheat reference genome (IWGSC RefSeq v1.0) according to the physical locations of flanking SNP markers. Strikingly, we identified cSNP‐harboured QTL for each of the agronomic traits by comparing the results of linkage mapping and GWAS (Supporting Information Table S10). Moreover, some of the QTL and co‐localized cSNPs were associated with multiple traits. For example, AX‐94964288, which was associated with GNP‐NS, GNP‐DS, GNP‐DHS, GYP‐NS, and GYP‐DS in GWAS, fell within QTL that related to GNP and GYP under both NS and DS. In addition, we also observed that a QTL on chromosome 2D was simultaneously related to SL and PH under both NS and DS; GWAS revealed that this QTL harboured a SL‐associated cSNP (AX‐108991361) and a PH‐associated cSNP (AX‐110276364); these two cSNPs are physically near (1.6 Mb) but not linked genetically (*r* ^2^ = 0.07; Figures 5a and 5b). Based on the variation of the two cSNPs, four haplotypes were identified in the germplasm population, that is, Collection 1. We observed that the PH and SL of *Hap*4 were significantly higher (longer) than that of accessions with *Hap*2 and *Hap*3, and much higher (longer) than that of accessions with *Hap*1 (Figure 5c), indicating that the alleles of these two cSNPs have additive effects on PH and SL. These results suggested that the causal genes controlling PH and SL on this QTL may not be single and further reflects higher resolution of GWAS in terms of genetic dissection compared with linkage mapping. Given the limited genetic diversity of biparental mapping population, previously reported QTL were also retrieved from the PubMed database; the physical location of these QTL were obtained from URGI databases and compared with our GWAS results. We found 23 cSNPs identified in the present study co‐localized with recently reported QTL for the same traits (Supporting Information Table S8). For instance, GNP‐NS‐associated cSNP (AX‐111073964) fell within a reported QTL (*QGns.cau‐2B.4*) controlling GNP (Guan et al., 2018). Moreover, our results showed that some of the QTL for agronomic traits under NS condition may also be effective under stressed environments. For example, IACX987 controlling TKW under NS condition (Ma, Xu, Ma, Li, & An, 2018) co‐localized with a cSNP (AX‐110931848) controlling TKW under both NS and DS. These results suggest that the cSNPs identified in the present study are reliable and enriched the information of reported QTL.

### 3.7 Haplotype blocks contain candidate genes controlling agronomic traits and/or stress tolerance

In total, 69.4% (206/295) of cSNPs identified in the present study fell within haplotype blocks (Supporting Information Table S8). We observed the Green Revolution dwarf gene *Rht‐D1* located on a haplotype block (4D, 17613616–18539635 bp), which harboured the PH‐associated cSNP (AX‐109665328), suggesting that predicting and annotating candidate genes on cSNP‐harbored haplotype blocks could be an effective method to identify causal genes. Therefore, the putative candidate genes in each cSNP‐harboured haplotype block were annotated and candidate genes were identified. The WRKY superfamily of transcription factors was reported to be involved in abiotic stress responses in wheat (Okay, Derelli, & Unver, 2014). We also noticed that three WRKY genes (*TaWRKY8*, *TaWRKY45*, and *TaWRKY70*) were co‐located on haplotype blocks with cSNPs. Also noteworthy, *TaWRKY8* located on the same haplotype block (6D, 104061501–104986040) with AX‐94471201, which was also associated with GYP‐DS‐R (3.41 × 10^−5^), GYP‐HS‐R (2.5 × 10^−3^), and GYP‐DHS‐R (3.48 × 10^−6^), suggesting that *TaWRKY8* may confer multiple abiotic stress tolerances. In addition, we also found that two haplotype blocks on chromosome 6A and 6D harboured AX‐94814333 and AX‐111021045, respectively, which were simultaneously associated with abiotic stress tolerance and GYP‐NS. Interestingly, only two (*TraesCS6A02G124100* and *TraesCS6A02G124200*) and one (*TraesCS6D02G114400*) genes were predicted in these two haplotype blocks, separately (Supporting Information Figure S3). Amongst, *TraesCS6A02G124100* and *TraesCS6D02G114400* are paralogous genes, which were annotated as putative ternary complex factor MIP1‐like. Rice *MIP1* (*MOC1 interacting protein*) exhibits pleiotropic phenotypes including enhanced tillering and yield (Sun et al., 2010), whereas the function of its wheat homolog was unknown. Moreover, we observed that the expression of *TraesCS6A02G124100* and *TraesCS6D02G114400* decreased after DS, HS, and DHS treatments in wheat on the basis of RNA‐Seq expression data from Wheat Expression Browser (http://www.wheat-expression.com/).

### 3.8 Selective sweep analysis

**Figure 6:**
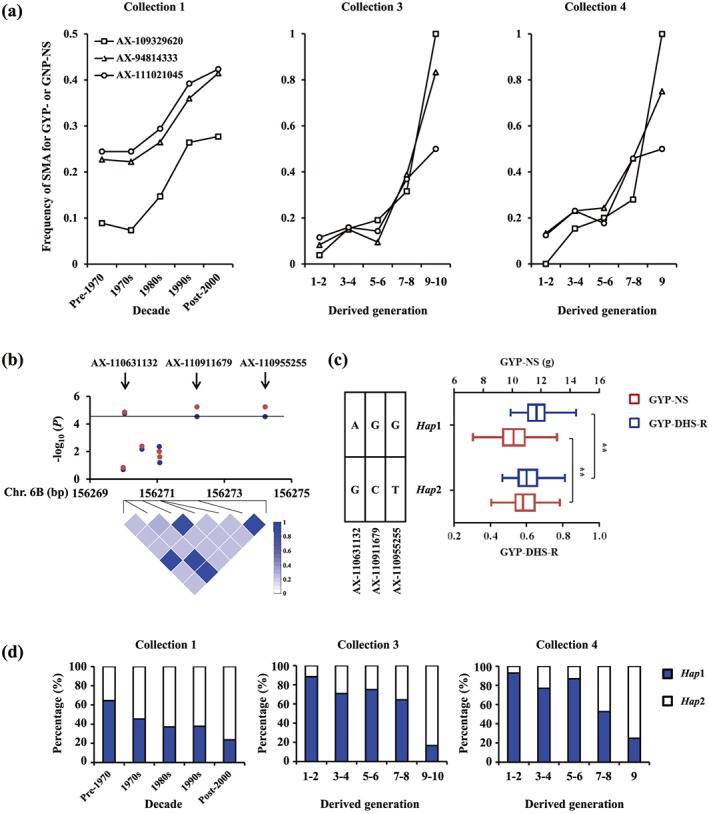
Trends of genetic variations of alleles or haplotypes (*Hap*) for tolerance‐production trade‐off along with decade or derived generation. (a) The frequency of superior marker allele (SMA) for grain yield per plant‐non‐stress (GYP‐NS; AX‐111021045 and AX‐94814333) or grain number per main spike‐non‐stress (GNP‐NS; AX‐109329620) increased along with decade or derived generation. (b) Local Manhattan plot (top) and linkage disequilibrium heatmap (bottom) surrounding the consistent candidate SNPs for GYP‐NS (AX‐110911679) and GYP‐DHS‐R (AX‐110631132) on chromosome 6B. (c) Boxplots for GYP‐NS and GYP‐DHS‐R based on the haplotypes (*Hap*), which formed by the variation of the marker allele in AX‐110911679, AX‐110631132, and AX‐110955255; different letters indicate statistically significant differences at the level of *p* < .01. (d) The percentage of accessions with different haplotypes for different eras or derived generations. Collection 1: 277 germplasm population; Collection 3: Xiaobaimai derivatives; Collection 4: Mazhamai derivatives [Colour figure can be viewed at http://wileyonlinelibrary.com]

Three genetic populations including 277 germplasm population (Collection 1), Xiaobaimai‐derived population (Collection 3), and Mazhamai‐derived population (Collection 4) were used to perform selective sweep analysis. For Collection 1, *F*_ST_ was calculated by contrasting cultivars released before 1970 and after 2000 (Supporting Information Table S1); for Collections 3 and 4, *F*_ST_ was calculated by contrasting cultivars of the first five derived generations and other derived generations (Supporting Information Table S2). Here, differentiated genomic regions with exceptionally large *F*_ST_ values (top 5%) were defined as selective sweep regions. For agronomic traits, 58 cSNPs fell within selective sweep regions for at least one collection, accounted for 30.7% (58/189) of the total cSNPs, suggesting that some of the favourable genetic variations in cSNPs were artificially selected in wheat breeding. However, only 23 cSNPs simultaneously fell within selective sweep region for two or three collections, among which 12 cSNPs were environment‐specific cSNPs, accounting for 7.6% (12/157) of the total environment‐specific cSNPs. Eleven cSNPs were consistent cSNPs (cSNPs associated with agronomic traits under different treatments), accounting for 34.3% (11/32) of the total consistent cSNPs suggesting that the favourable variations of consistent cSNPs are more likely to be selected in different breeding process when compared with that of environment specific cSNPs. For abiotic stress tolerances, only 10 cSNPs fell within selective sweep regions in at least one population; moreover, four of the cSNPs, that is, AX‐111021045, AX‐94814333, AX‐109329620, and AX‐110631132, simultaneously related to agronomic traits with adverse allelic effect. To be specific, AX‐111021045 and AX‐94814333 are the common cSNPs for GYP‐NS, GYP‐DS‐R, GYP‐HS‐R, and GYP‐DHS‐R, which were mentioned above that co‐located with putative *MIP1*‐like genes on 6A and 6D, and AX‐109329620 is the common cSNP for GNP‐NS and GNP‐HS‐R. We observed that the frequency of superior marker alleles (SMAs) for GYP‐NS or GNP‐NS increased sharply along with the decade or derived generation (Figure 6a); accordingly, another allele‐type conferring high stress tolerance decreased. Furthermore, AX‐110631132 is the cSNP for GYP‐DHS‐R and co‐located on a haplotype block (6B, 156269992–15627421 bp) with GYP‐NS‐associated cSNP (AX‐110911679); in total, three SNPs exceed the threshold value in the haplotype block (Figure 6b). Based on the variations of the three SNPs, two haplotypes (*Hap*1 and *Hap*2) were identified due to the close linkage among the three SNPs. *Hap*1 presented high YP‐DHS‐R but low GYP‐NS, whereas *Hap*2 exhibited low YP‐DHS‐R but high GYP‐NS (Figure 6c). We also observed that the frequency of *Hap*1 decreased along with breeding process, whereas *Hap*2 increased (Figure 6d). What is more, the variational pattern of alleles or haplotypes is consistent with the chronological variation of agronomic traits and stress tolerances. For instance, the average GYP‐NS gradually increase from 9.8 g for cultivars released before 1970 to 11.0 g for cultivars released after 2000, whereas the average GYP‐DS‐R, GYP‐HS‐R, and GYP‐DHS‐R gradually decrease from 0.87, 0.84, and 0.68 for cultivars released before 1970 to 0.83, 0.80, and 0.64 for cultivars released after 2000, separately (Supporting Information Figure S4).

## 4 DISCUSSION

Wheat plays a crucial role in sustaining food security but greatly threatened by drought and heat stresses worldwide. To gain insights about genetic architecture and candidate genes underlying high yield and wide adaptation of wheat, we investigated the genotypic variability for drought and heat‐responsive agronomic traits under multiple environments and conducted GWAS and linkage mapping using high‐density wheat 660K SNP array. To the best of our knowledge, several findings of this study are novel. Firstly, 295 associated loci including consistent loci for agronomic traits across different environments and eurytopic loci for drought and heat stress tolerances were identified and anchored on newly released wheat reference genome with exact physical locations, and cSNP‐harboured QTL for each of the agronomic traits were identified by comparing the results of linkage mapping and GWAS; these information further clarified the genomic regions controlling yield‐related agronomic traits and combined drought, and heat stress tolerance in wheat will be of great value for scientists and breeders to discover functional genes and realize molecular breeding in times of unexpected climatic fluctuations. Secondly, selective sweep analysis showed that the gradual loss of advantageous variations for abiotic stress tolerances due to preferential selection for yield potential may be a common phenomenon in current wheat breeding. More importantly, two promising candidate paralogous genes *TraesCS6A02G124100* and *TraesCS6D02G114400* controlling GYP and multiple stress tolerance also open up an interesting and novel field of investigation on the mechanisms underlying the stress tolerance–productivity trade‐off in wheat. These key findings from our study are further discussed below.

### 4.1 Preparatory works to perform GWAS for drought and heat stress tolerances in wheat

It is often difficult to perform GWAS for drought and heat stress tolerances at later growth periods owing to two major reasons. The first is that diversity panels of crop species often have diverse phenology, whose flowering time in the field likely varies by a long duration (Kadam, Struik, Rebolledo, Yin, & Jagadish, 2018; Pinto et al., 2010). Although statistical adjustment was used to address this problem (Sukumaran et al., 2018), it must be noted that the confounding effect of flowering time may not be completely controlled, and the artificial adjustment of phenotypic value may decrease the validity of GWAS results. Therefore, based on previous records, we selected 277 accessions (Collection 1) with similar phenology for GWAS, most of whose flowering dates occurred within 1 week. The second difficulty is implementing heat stress under field condition; staggered sowing is an effective strategy widely used in previous studies (Guan et al., 2018), but it may have confounding effects on other factors, such as illumination intensity and rainfall cross the whole growth period. To deal with this issue, we installed large‐scale steel frames over the trail plots before sowing and aggravated terminal heat stress by covering the steel frames with transparent plastic film 1‐week post‐anthesis. Moreover, we planted guard rows around each trail plot to decrease border effects. Although these strategies still have some disadvantages, for example, air humidity inside the shed increased about 10 to 20% due to the poor ventilation conditions, our results indicate value for simulating heat studies.

### 4.2 Wheat should be studied under combined drought and heat stress to improve dual tolerance

Drought and heat are considered the two major abiotic stresses constraining global wheat production (Barnabas, Jaeger, & Feher, 2008). Although wheat responses to drought and heat stresses are complex and share some common mechanisms, the combination of drought and heat has an additive negative impact on wheat growth and development; for example, drought or heat alone reduce leaf chlorophyll content by 9% or 27% and combined drought and heat stress decreases leaf chlorophyll content by 49% (Awasthi et al., 2014). Consistently, the lowest BLUPs of all agronomic traits were observed under DHS in our study. It is possibly because the combined drought and heat stress alter plant metabolism in novel ways compared with single stresses (Zandalinas, Mittler, Balfagon, Arbona, & Gomez‐Cadenas, 2018). *Arabidopsis*, subjected to combined drought and heat stress, revealed a new pattern of stress tolerance, as well as 454 transcripts, which were specifically expressed when the dual stress occurred (Rizhsky et al., 2004). However, very little is known about the specific molecular mechanisms underlying crop tolerance to the combined heat and drought stress (Tricker et al., 2018). Our study identified numerous allelic variations specifically controlling drought tolerance or heat tolerance while not associated with combined drought and heat tolerance and vice versa, suggesting that wheat should be studied under combined stress to improve dual tolerance. Moreover, the allelic variations controlling tolerance to combined drought and heat stress detected in our study may be valuable in crop research, helping discovery of functional genes and in developing cultivars with resilience to future climate change scenarios.

### 4.3 Consistent marker–trait associations

Many agronomic traits are complex quantitative characteristics affected by environment; we observed that most of the marker–trait associations were specific to NS or stress conditions, as observed in previous studies (Kadam et al., 2018), suggesting that environment specificity is likely to be a common characteristic of QTL. Nevertheless, we also observed consistent marker–trait associations, and some of them were also detected by biparental QTL mapping. Moreover, some reported QTL identified in different populations overlapped with the current results. For instance, a QTL controlling PH and SL were identified by GWAS and biparental QTL mapping in the present study (Figures 5a and 5b). This QTL co‐located with a QTL that was reported in a F_2:3_ population derived from the cross of H164 × Sumai3, of which H164 was a dwarf and compact spike mutant obtained from Sumai3 treated with ethyl methane sulfonate (Gao, 2009). Moreover, according to the results of LD and haplotype analysis, we speculate that more than one causal gene may be controlling PH and SL on this QTL. These results indicated that our GWAS results are reliable and contained supplementary information for the reported QTL; the consistent marker–trait associations will accelerate molecular breeding in wheat.

### 4.4 Promising candidate genes for stress tolerance–productivity trade‐off in wheat

Better understanding of trade‐off between productivity or yield potential and abiotic stress tolerance is essential for crop improvement. Relevant molecular mechanisms have been revealed in *Arabidopsis* (Han et al., 2012), but seldom in studies for major crops. We detected two putative *MIP1‐like* genes, that is, *TraesCS6A02G124100* (6A) and *TraesCS6D02G114400* (6D), near cSNPs within the haplotype blocks for GYP‐NS and abiotic stress tolerances (Supporting Information Figure S3). In rice, *MIP1* interacted with *MONOCULM1* (*MOC1*, a major gene controlling the formation of tiller buds), and the overexpression of *MIP1* resulted in enhanced tillering and grain yield (Sun et al., 2010). Therefore, we hypothesized that *TraesCS6A02G124100* and *TraesCS6D02G114400* may similarly enhanced grain yield in wheat. Furthermore, RNA sequencing indicated that the expression of *TraesCS6A02G124100* and *TraesCS6D02G114400* both decreased after DS, HS, and DHS treatments. Therefore, further studies using molecular biology methods on these genes could help to explain the molecular mechanism of the trade‐off between yield potential and abiotic stress tolerance in crops.

### 4.5 Current wheat breeding pattern should be optimized for sustainable agriculture in China

Chinese agriculture has intensified over the decades with large inputs of resources, that is, water and fertilizers, and improving yield potential is a major objective for breeders (Guo et al., 2010). Our results suggest that the genetic variation controlling yield‐related traits under NS condition are more likely to be selected in breeding (Supporting Information Table S8). However, strong selection for genetic variation controlling yield‐related traits may diminish genetic variation for traits influencing stress tolerance due to heritable covariation (Voss‐Fels et al., 2017). Here, we identified six loci simultaneously associated with yield‐related traits and abiotic stress tolerances; four of them fell within selective sweep regions. The frequency of SMA for yield‐related traits in the four loci increased in three different populations along with decade or generation, whereas decreased for SMA controlling abiotic stress tolerance. These results suggest that the gradual loss of advantageous variation for abiotic stress tolerances due to preferential selection for yield potential may be a common phenomenon in current wheat breeding. Therefore, this breeding pattern should be revised by conscious selection for sustainable yields across a wider range of likely climate variation. Strategic molecular breeding is a promising strategy to address multiple selection criteria (Zhang, Massel, Godwin, & Gao, 2018) but requires sufficient information about stable genetic variation and further understanding trait covariation and interaction. In conclusion, the results of present study are timely in terms of developing efficient molecular markers and dissection of the functions of relevant genes.

## CONFLICT OF INTERESTS

The authors have no competing interests.

## Supporting information


**Figure S1.** Trial plots for drought stress (a) and heat stress (b)

**Figure S2.** The air temperature and soil water content under different environments.

**Figure S3.** The physical position of haplotype blocks which harbored candidate genes (TraesCS6A02G124100 and TraesCS6D02G114400) and cSNPs for tolerance‐production trade‐off.

**Figure S4.** The chronological variation of agronomic traits and stress tolerances

**Table S1.** Information of 277 wheat accessions
**Table S2.** Cultivars derived from Xiaobaimai and Mazhamai
**Table S3.** Information of investigated traits and precipitation in each year and site
**Table S4.** Statistical information of linkage map for DH population
**Table S5.** Analysis of variance (ANOVA) of agronomic traits components, and other related traits
**Table S6.** Summary statistics of BLUPs of agronomic traits
**Table S7.** Number of SNPs, haplotypes and haplotype blocks on each chromosome
**Table S8.** Information of candidate SNPs (cSNPs) detected by GWAS
**Table S9.** Information of QTL detected by linkage mapping
**Table S10.** Summary of cSNP‐harbored QTL

## References

[pce13577-bib-0001] Awasthi, R. , Kaushal, N. , Vadez, V. , Turner, N. C. , Berger, J. , Siddique, K. H. M. , & Nayyar, H. (2014). Individual and combined effects of transient drought and heat stress on carbon assimilation and seed filling in chickpea. Functional Plant Biology, 41, 1148–1167. 10.1071/FP13340 32481065

[pce13577-bib-0002] Barnabas, B. , Jaeger, K. , & Feher, A. (2008). The effect of drought and heat stress on reproductive processes in cereals. Plant, Cell & Environment, 31, 11–38. 10.1111/j.1365-3040.2007.01727.x 17971069

[pce13577-bib-0003] Cui, F. , Zhang, N. , Fan, X. , Zhang, W. , Zhao, C. , Yang, L. , … Li, J. (2017). Utilization of a Wheat660K SNP array‐derived high‐density genetic map for high‐resolution mapping of a major QTL for kernel number. Scientific Reports, 7, 3788 10.1038/s41598-017-04028-6 28630475 PMC5476560

[pce13577-bib-0004] Dai, A. (2013). Increasing drought under global warming in observations and models. Nature Climate Change, 3, 52–58. 10.1038/nclimate1633

[pce13577-bib-0005] Fang, C. , Ma, Y. , Wu, S. , Liu, Z. , Wang, Z. , Yang, R. , … Tian, Z. (2017). Genome‐wide association studies dissect the genetic networks underlying agronomical traits in soybean. Genome Biology, 18, 161 10.1186/s13059-017-1289-9 28838319 PMC5571659

[pce13577-bib-0006] Gabriel, S. B. , Schaffner, S. F. , Nguyen, H. , Moore, J. M. , Roy, J. , Blumenstiel, B. , … Altshuler, D. (2002). The structure of haplotype blocks in the human genome. Science, 296, 2225–2229. 10.1126/science.1069424 12029063

[pce13577-bib-0007] Gao, R. H. (2009). Genetic analysis and QTL mapping of dwarf and compact spike mutant of Sumai3. Nanjing, China: MS Thesis of Nanjing Agricultural University. (in Chinese with English abstract)

[pce13577-bib-0008] Godfray, H. C. J. , Beddington, J. R. , Crute, I. R. , Haddad, L. , Lawrence, D. , Muir, J. F. , … Toulmin, C. (2010). Food security: The challenge of feeding 9 billion people. Science, 327, 812–818. 10.1126/science.1185383 20110467

[pce13577-bib-0009] Guan, P. , Lu, L. , Jia, L. , Kabir, M. R. , Zhang, J. , Lan, T. , … Peng, H. (2018). Global QTL analysis identifies genomic regions on chromosomes 4A and 4B harboring stable loci for yield‐related traits across different environments in wheat (*Triticum aestivum* L.). Frontiers in Plant Science, 9, 529 10.3389/fpls.2018.00529 29922302 PMC5996883

[pce13577-bib-0010] Guo, J. , Liu, X. , Zhang, Y. , Shen, J. , Han, W. , Zhang, W. , … Zhang, F. (2010). Significant acidification in major Chinese croplands. Science, 327, 1008–1010. 10.1126/science.1182570 20150447

[pce13577-bib-0011] Guo, Z. , Yang, W. , Chang, Y. , Ma, X. , Tu, H. , Xiong, F. , … Xiong, L. (2018). Genome‐wide association studies of image traits reveal genetic architecture of drought resistance in rice. Molecular Plant, 11, 789–805. 10.1016/j.molp.2018.03.018 29614319

[pce13577-bib-0012] Han, S. , Sang, Y. , Rodrigues, A. , Wu, M. , Rodriguez, P. L. , Wagner, D. , & Biol, F. (2012). The SWI2/SNF2 chromatin remodeling ATPase BRAHMA represses abscisic acid responses in the absence of the stress stimulus in *Arabidopsis* . Plant Cell, 24, 4892–4906. 10.1105/tpc.112.105114 23209114 PMC3556964

[pce13577-bib-0013] Hao, Z. , Chang, X. , Guo, X. , Jing, R. , Li, R. , & Jia, J. (2003). QTL mapping for drought tolerance at stages of germination and seedling in wheat (*Triticum aestivum* L.) using a DH population. Agricultural Sciences in China, 2, 943–949.

[pce13577-bib-0014] International Wheat Genome Sequencing Consortium (IWGSC) (2018). Shifting the limits in wheat research and breeding using a fully annotated reference genome. Science, 361, eaar7191. 30115783 10.1126/science.aar7191

[pce13577-bib-0015] Kadam, N. N. , Struik, P. C. , Rebolledo, M. C. , Yin, X. , & Jagadish, S. V. K. (2018). Genome‐wide association reveals novel genomic loci controlling rice grain yield and its component traits under water‐deficit stress during the reproductive stage. Journal of Experimental Botany, 69, 4017–4032. 10.1093/jxb/ery186 29767744 PMC6054195

[pce13577-bib-0016] Kulkarni, M. , Soolanayakanahally, R. , Ogawa, S. , Uga, Y. , Selvaraj, M. G. , & Kagale, S. (2017). Drought response in wheat: key genes and regulatory mechanisms controlling root system architecture and transpiration efficiency. Frontiers in Chemistry, 5, 106 10.3389/fchem.2017.00106 29259968 PMC5723305

[pce13577-bib-0017] Langridge, P. , & Reynolds, M. P. (2015). Genomic tools to assist breeding for drought tolerance. Current Opinion in Biotechnology, 32, 130–135. 10.1016/j.copbio.2014.11.027 25531270

[pce13577-bib-0018] Li, H. , Ribaut, J. M. , Li, Z. , & Wang, J. (2008). Inclusive composite interval mapping (ICIM) for digenic epistasis of quantitative traits in biparental populations. Theoretical and Applied Genetics, 116, 243–260. 10.1007/s00122-007-0663-5 17985112

[pce13577-bib-0019] Li, M. X. , Yeung, J. M. Y. , Cherny, S. S. , & Sham, P. C. (2012). Evaluating the effective numbers of independent tests and significant p‐value thresholds in commercial genotyping arrays and public imputation reference datasets. Human Genetics, 131, 747–756. 10.1007/s00439-011-1118-2 22143225 PMC3325408

[pce13577-bib-0020] Liu, C. , Sivakumar, S. , Etienne, C. , Carolina, S. , Susanne, D. , & Matthew, R. (2019). Genetic dissection of heat and drought stress QTLs in phenology‐controlled synthetic‐derived recombinant inbred lines in spring wheat. Molecular Breeding, 39, 34 10.1007/s11032-019-0938-y

[pce13577-bib-0021] Liu, X. , Huang, M. , Fan, B. , Buckler, E. S. , & Zhang, Z. (2016). Iterative usage of fixed and random effect models for powerful and efficient genome‐wide association studies. PLoS Genetics, 12, e1005767 10.1371/journal.pgen.1005767 26828793 PMC4734661

[pce13577-bib-0022] Liu, X. , Li, R. , Chang, X. , & Jing, R. (2013). Mapping QTLs for seedling root traits in a doubled haploid wheat population under different water regimes. Euphytica, 189, 51–66. 10.1007/s10681-012-0690-4

[pce13577-bib-0023] Lobell, D. B. , Sibley, A. , & Ivan Ortiz‐Monasterio, J. (2012). Extreme heat effects on wheat senescence in India. Nature Climate Change, 2, 186–189. 10.1038/nclimate1356

[pce13577-bib-0024] Ma, F. , Xu, Y. , Ma, Z. , Li, L. , & An, D. (2018). Genome‐wide association and validation of key loci for yield‐related traits in wheat founder parent Xiaoyan 6. Molecular Breeding, 38, 91 10.1007/s11032-018-0837-7

[pce13577-bib-0025] Maulana, F. , Ayalew, H. , Anderson, J. D. , Kumssa, T. T. , Huang, W. , & Ma, X. (2018). Genome‐wide association mapping of seedling heat tolerance in winter wheat. Frontiers in Plant Science, 9, 1272 10.3389/fpls.2018.01272 30233617 PMC6131858

[pce13577-bib-0026] Mora, F. , Castillo, D. , Lado, B. , Matus, I. , Poland, J. , Belzile, F. , … del Pozo, A. (2015). Genome‐wide association mapping of agronomic traits and carbon isotope discrimination in a worldwide germplasm collection of spring wheat using SNP markers. Molecular Breeding, 35, 69 10.1007/s11032-015-0264-y

[pce13577-bib-0027] Nevo, E. , & Chen, G. (2010). Drought and salt tolerances in wild relatives for wheat and barley improvement. Plant, Cell & Environment, 33, 670–685. 10.1111/j.1365-3040.2009.02107.x 20040064

[pce13577-bib-0028] Ogbonnaya, F. C. , Seah, S. , Delibes, A. , Jahier, J. , López‐Braña, I. , Eastwood, R. F. , & Lagudah, E. S. (2001). Molecular‐genetic characterisation of a new nematode resistance gene in wheat. Theoretical and Applied Genetics, 102, 623–629. 10.1007/s001220051689

[pce13577-bib-0029] Okay, S. , Derelli, E. , & Unver, T. (2014). Transcriptome‐wide identification of bread wheat WRKY transcription factors in response to drought stress. Molecular Genetics and Genomics, 289, 765–781. 10.1007/s00438-014-0849-x 24748053

[pce13577-bib-0030] Olesen, J. E. , Trnka, M. , Kersebaum, K. C. , Skjelvag, A. O. , Seguin, B. , Peltonen‐Sainio, P. , … Micale, F. (2011). Impacts and adaptation of European crop production systems to climate change. European Journal of Agronomy, 34, 96–112. 10.1016/j.eja.2010.11.003

[pce13577-bib-0031] Pace, J. , Gardner, C. , Romay, C. , Ganapathysubramanian, B. , & Luebberstedt, T. (2015). Genome‐wide association analysis of seedling root development in maize (*Zea mays* L.). BMC Genomics, 16, 47 10.1186/s12864-015-1226-9 25652714 PMC4326187

[pce13577-bib-0032] Pask, A. J. D. , Pietragalla, J. , Mullan, D. M. , & Reynolds, M. P. (2012). Physiological breeding II: a field guide to wheat phenotyping In Pietragalla J., & Pask A. (Eds.), Grain yield and yield components (pp. 95–103). Mexico: CIMMYT.

[pce13577-bib-0033] Piepho, H. P. , & Moehring, J. (2007). Computing heritability and selection response from unbalanced plant breeding trials. Genetics, 177, 1881–1888. 10.1534/genetics.107.074229 18039886 PMC2147938

[pce13577-bib-0034] Pinto, R. S. , Reynolds, M. P. , Mathews, K. L. , McIntyre, C. L. , Olivares‐Villegas, J. J. , & Chapman, S. C. (2010). Heat and drought adaptive QTL in a wheat population designed to minimize confounding agronomic effects. Theoretical and Applied Genetics, 121, 1001–1021. 10.1007/s00122-010-1351-4 20523964 PMC2938441

[pce13577-bib-0035] Pradhan, G. P. , Prasad, P. V. V. , Fritz, A. K. , Kirkham, M. B. , & Gill, B. S. (2012). Effects of drought and high temperature stress on synthetic hexaploid wheat. Functional Plant Biology, 39, 190–198. 10.1071/FP11245 32480773

[pce13577-bib-0036] Prasad, P. V. V. , Pisipati, S. R. , Momcilovic, I. , & Ristic, Z. (2011). Independent and combined effects of high temperature and drought stress during grain filling on plant yield and chloroplast EF‐Tu expression in spring wheat. Journal of Agronomy and Crop Science, 197, 430–441. 10.1111/j.1439-037X.2011.00477.x

[pce13577-bib-0037] Qian, Q. , Guo, L. , Smith, S. M. , & Li, J. (2016). Breeding high‐yield superior quality hybrid super rice by rational design. National Science Review, 3, 283–294. 10.1093/nsr/nww006

[pce13577-bib-0038] Ray, D. K. , Mueller, N. D. , West, P. C. , & Foley, J. A. (2013). Yield trends are insufficient to double global crop production by 2050. PLoS ONE, 8, e66428 10.1371/journal.pone.0066428 23840465 PMC3686737

[pce13577-bib-0039] Rizhsky, L. , Liang, H. J. , Shuman, J. , Shulaev, V. , Davletova, S. , & Mittler, R. (2004). When defense pathways collide. The response of *Arabidopsis* to a combination of drought and heat stress. Plant Physiology, 134, 1683–1696. 10.1104/pp.103.033431 15047901 PMC419842

[pce13577-bib-0040] Sakuma, Y. , Maruyama, K. , Qin, F. , Osakabe, Y. , Shinozaki, K. , & Yamaguchi‐Shinozaki, K. (2006). Dual function of an Arabidopsis transcription factor DREB2A in water‐stress‐responsive and heat‐stress‐responsive gene expression. Proceedings of the National Academy of Sciences of the United States of America, 103, 18822–18827. 10.1073/pnas.0605639103 17030801 PMC1693746

[pce13577-bib-0041] Schulthess, A. W. , Reif, J. C. , Ling, J. , Plieske, J. , Kollers, S. , Ebmeyer, E. , … Jiang, Y. (2017). The roles of pleiotropy and close linkage as revealed by association mapping of yield and correlated traits of wheat (*Triticum aestivum* L.). Journal of Experimental Botany, 68, 4089–4101. 10.1093/jxb/erx214 28922760 PMC5853857

[pce13577-bib-0042] Sukumaran, S. , Dreisigacker, S. , Lopes, M. , Chavez, P. , & Reynolds, M. P. (2015). Genome‐wide association study for grain yield and related traits in an elite spring wheat population grown in temperate irrigated environments. Theoretical and Applied Genetics, 128, 353–363. 10.1007/s00122-014-2435-3 25490985

[pce13577-bib-0043] Sukumaran, S. , Reynolds, M. P. , & Sansaloni, C. (2018). Genome‐wide association analyses identify QTL hotspots for yield and component traits in durum wheat grown under yield potential, drought, and heat stress environments. Frontiers in Plant Science, 9, 81 10.3389/fpls.2018.00081 29467776 PMC5808252

[pce13577-bib-0044] Sun, C. , Zhang, F. , Yan, X. , Zhang, X. , Dong, Z. , Cui, D. , & Chen, F. (2017). Genome‐wide association study for 13 agronomic traits reveals distribution of superior alleles in bread wheat from the Yellow and Huai Valley of China. Plant Biotechnology Journal, 15, 953–969. 10.1111/pbi.12690 28055148 PMC5506658

[pce13577-bib-0045] Sun, F. , Zhang, W. , Xiong, G. , Yan, M. , Qian, Q. , Li, J. , & Wang, Y. (2010). Identification and functional analysis of the MOC1 interacting protein 1. Journal of Genetics and Genomics, 37, 69–77. 10.1016/S1673-8527(09)60026-6 20171579

[pce13577-bib-0046] Suzuki, N. , Rivero, R. M. , Shulaev, V. , Blumwald, E. , & Mittler, R. (2014). Abiotic and biotic stress combinations. New Phytologist, 203, 32–43. 10.1111/nph.12797 24720847

[pce13577-bib-0047] Tilman, D. , Balzer, C. , Hill, J. , & Befort, B. L. (2011). Global food demand and the sustainable intensification of agriculture. Proceedings of the National Academy of Sciences of the United States of America, 108, 20260–20264. 10.1073/pnas.1116437108 22106295 PMC3250154

[pce13577-bib-0048] Tricker, P. J. , Elhabti, A. , Schmidt, J. , & Fleury, D. (2018). The physiological and genetic basis of combined drought and heat tolerance in wheat. Journal of Experimental Botany, 69, 3195–3210. 10.1093/jxb/ery081 29562265

[pce13577-bib-0049] Voss‐Fels, K. P. , Qian, L. , Parra‐Londono, S. , Uptmoor, R. , Frisch, M. , Keeble‐Gagnere, G. , … Snowdon, R. J. (2017). Linkage drag constrains the roots of modern wheat. Plant, Cell & Environment, 40, 717–725. 10.1111/pce.12888 28036107

[pce13577-bib-0050] Wen, Y. , Zhang, H. , Ni, Y. , Huang, B. , Zhang, J. , Feng, J. , … Wu, R. (2018). Methodological implementation of mixed linear models in multi‐locus genome‐wide association studies. Briefings in Bioinformatics, 19, 700–712. 28158525 10.1093/bib/bbw145 PMC6054291

[pce13577-bib-0051] Yano, K. , Yamamoto, E. , Aya, K. , Takeuchi, H. , Lo, P. , Hu, L. , … Matsuoka, M. (2016). Genome‐wide association study using whole‐genome sequencing rapidly identifies new genes influencing agronomic traits in rice. Nature Genetics, 48, 927–934. 10.1038/ng.3596 27322545

[pce13577-bib-0052] Zandalinas, S. I. , Mittler, R. , Balfagon, D. , Arbona, V. , & Gomez‐Cadenas, A. (2018). Plant adaptations to the combination of drought and high temperatures. Physiologia Plantarum, 162, 2–12. 10.1111/ppl.12540 28042678

[pce13577-bib-0053] Zhang, Y. , Massel, K. , Godwin, I. D. , & Gao, C. (2018). Applications and potential of genome editing in crop improvement. Genome Biology, 19 10.1186/s13059-018-1586-y PMC6267055 30501614

