# Supplementary figures and images for: Genetic dissection of drought and heat‐responsive agronomic traits in wheat
Source: Plant Cell Environ. 2019 Jun 24;42(9):2540–53. doi: 10.1111/pce.13577 (PMC6851630; doi:10.1111/pce.13577)

(a)

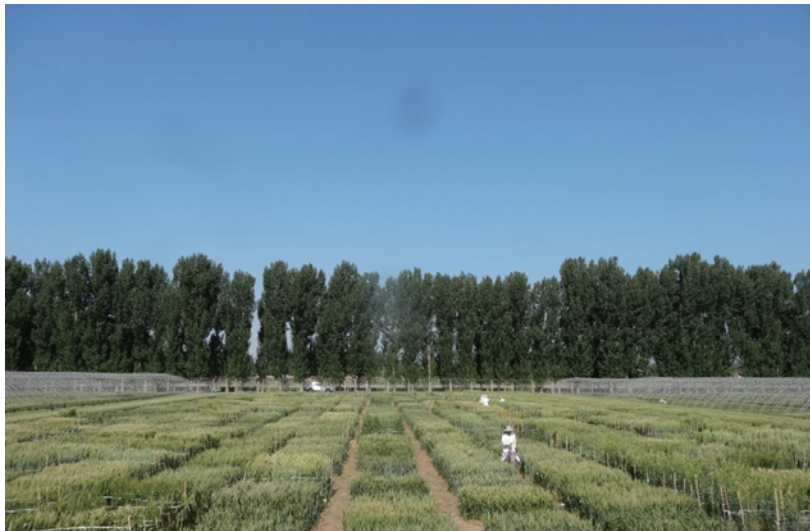

(b)

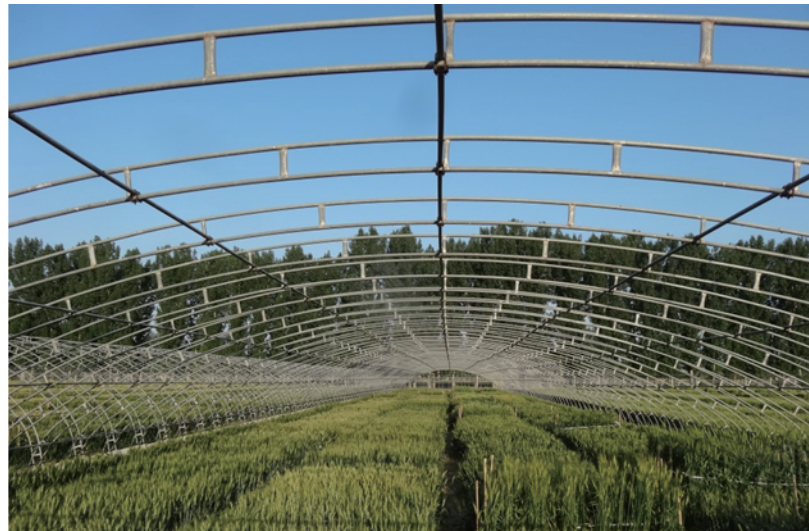

Fig. S1. Trial plots for drought stress (a) and heat stress (b)

Supplement: Supplementary file 1 — Figure S1. Trial plots for drought stress (a) and heat stress (b) [file PCE-42-2540-s001.pdf]

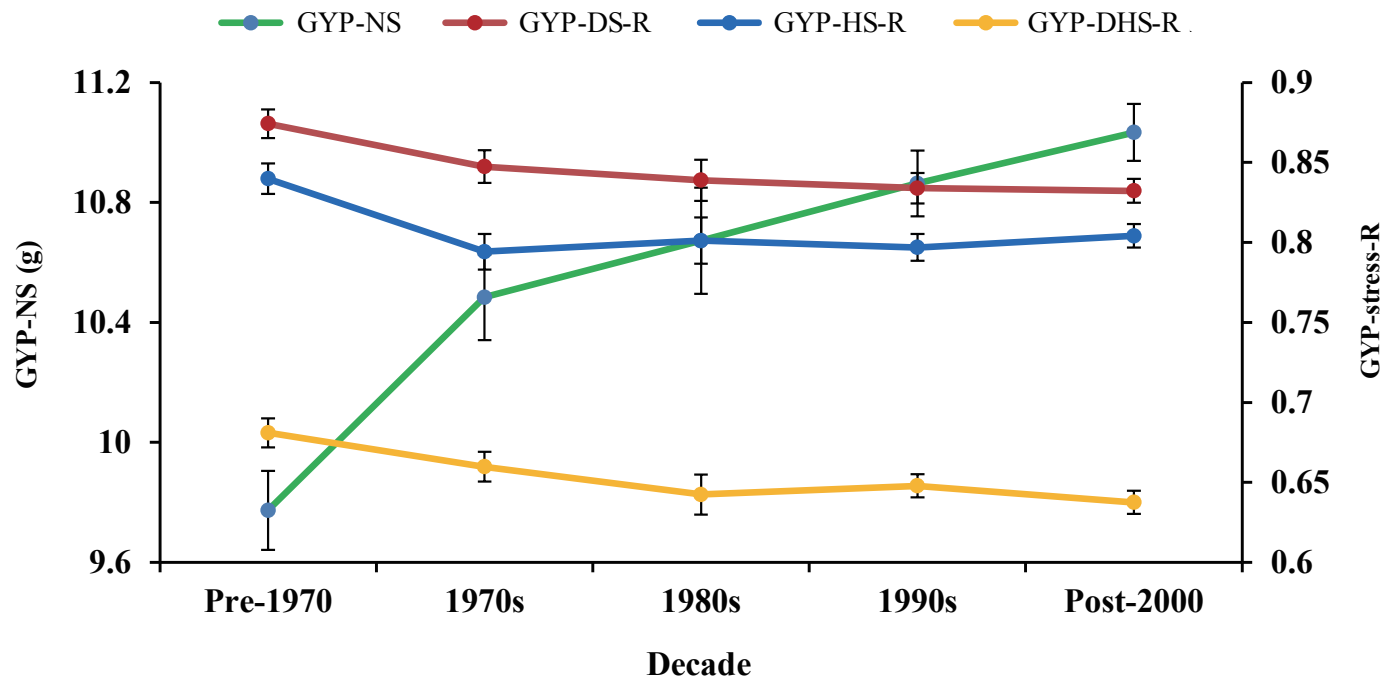

Fig.S4 The chronological variation of agronomic traits and stress tolerances

Supplement: Supplementary file 4 — Figure S4. The chronological variation of agronomic traits and stress tolerances [file PCE-42-2540-s004.pdf]
