# Supplementary material for: Genetic dissection of drought and heat‐responsive agronomic traits in wheat
Source: Plant Cell Environ. 2019 Jun 24;42(9):2540–53. doi: 10.1111/pce.13577 (PMC6851630; doi:10.1111/pce.13577)
Supplement: Supplementary file 2 — Figure S2. The air temperature and soil water content under different environments. [file PCE-42-2540-s002.pdf]

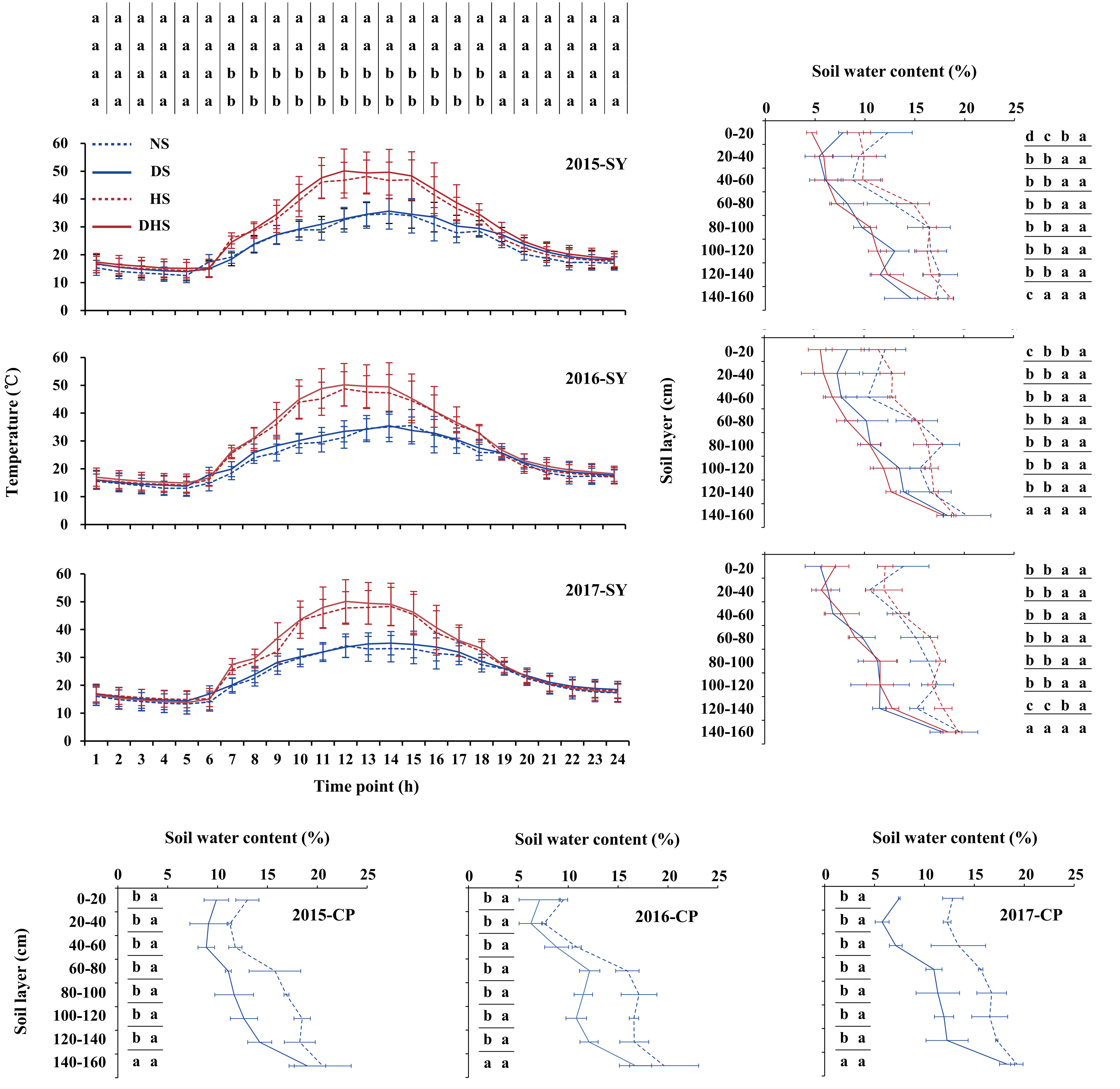

Fig. S2. The air temperature and soil water content under different environments.
CP: Changping, SY: Shunyi. Different letters indicate statistically significant differences for the corresponding points at the level of *P* < 0.01.
