# Supplementary material for: Genetic dissection of drought and heat‐responsive agronomic traits in wheat
Source: Plant Cell Environ. 2019 Jun 24;42(9):2540–53. doi: 10.1111/pce.13577 (PMC6851630; doi:10.1111/pce.13577)
Supplement: Supplementary file 3 — Figure S3. The physical position of haplotype blocks which harbored candidate genes (TraesCS6A02G124100 and TraesCS6D02G114400) and cSNPs for tolerance‐production trade‐off. [file PCE-42-2540-s003.pdf]

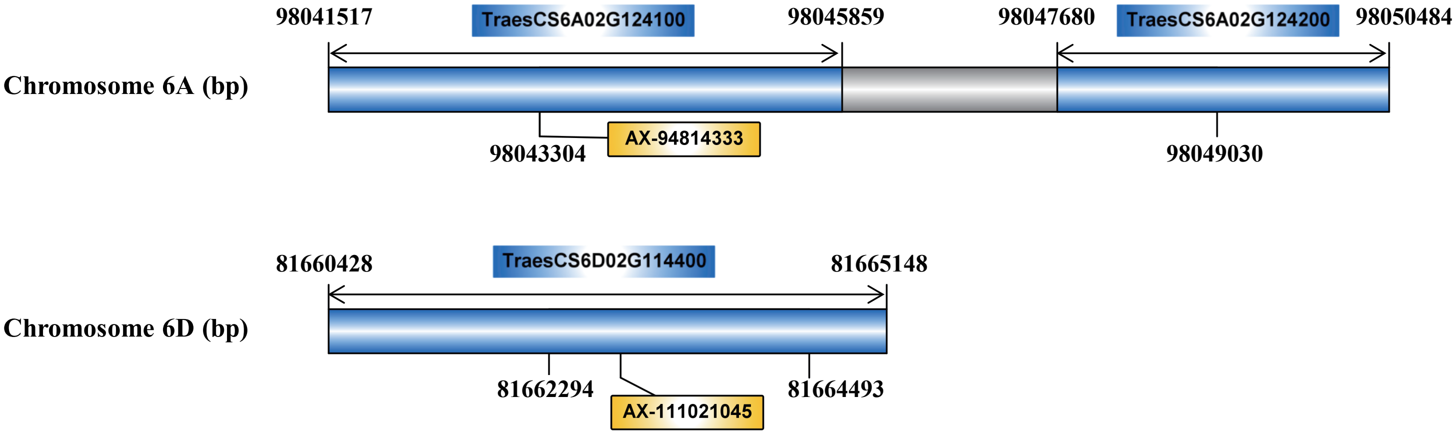

Fig. S3. The physical position of haplotype blocks which harbored candidate genes (*TraesCS6A02G124100* and *TraesCS6D02G114400*) and cSNPs for tolerance-production trade-off.
